
JOURNAL INFORMATION
==============================
NLM Title Abbreviation: Biospektrum (Heidelb)
Journal ID: Biospektrum (Heidelb)
NLM Journal ID: 9615685

Biospektrum

ISSN: 0947-0867
EISSN: 1868-6249

ARTICLE INFORMATION
==============================
PMCID: PMC7880628
DOI: 10.1007/s12268-021-1519-3
Article ID: 1519
Article version: 1
Subjects: Karriere, Köpfe & Konzepte

Buchrezension zu: Das Gesetz der Ansteckung

Was Pandemien, Börsencrashs und Fake News gemeinsam haben

Mansmann Ulrich mansmann@ibe.med.uni-muenchen.de

grid.5252.0 0000 0004 1936 973X Universität München, München, Deutschland
Electronic publication date: 2021 Feb 12
Print publication date: 2021
Volume: 27
Issue: 1
First page: 109
Last page: 109
Copyright: © Der Autor 2021
License: Dieser Artikel wird unter der Creative Commons Namensnennung 4.0 International Lizenz veröffentlicht, welche die Nutzung, Vervielfältigung, Bearbeitung, Verbreitung und Wiedergabe in jeglichem Medium und Format erlaubt, sofern Sie den/die ursprünglichen Autor(en) und die Quelle ordnungsgemäß nennen, einen Link zur Creative Commons Lizenz beifügen und angeben, ob Änderungen vorgenommen wurden. Die in diesem Artikel enthaltenen Bilder und sonstiges Drittmaterial unterliegen ebenfalls der genannten Creative Commons Lizenz, sofern sich aus der Abbildungslegende nichts anderes ergibt. Sofern das betreffende Material nicht unter der genannten Creative Commons Lizenz steht und die betreffende Handlung nicht nach gesetzlichen Vorschriften erlaubt ist, ist für die oben aufgeführten Weiterverwendungen des Materials die Einwilligung des jeweiligen Rechteinhabers einzuholen. Weitere Details zur Lizenz entnehmen Sie bitte der Lizenzinformation auf http://creativecommons.org/licenses/by/4.0/deed.de.
License URL: https://creativecommons.org/licenses/by/4.0/

issue-copyright-statement: © Springer-Verlag GmbH Deutschland, ein Teil von Springer Nature 2021

==============================

*339 S., 28 Abb., Hirzel, 2020. HC, 26,– €.*
*ISBN: 9783777629049*
*Auch als E-Book erhältlich*

■ Adam Kucharski beschreibt die Entwicklung infektionsepidemiologischer Konzepte und deren Interaktion mit anderen Disziplinen (Beherrschung von Finanzkrisen, Bewältigung von Gewaltausbrüchen, Lenkung von Inhalten im Internet, Sprachentwicklung, kulturelle Lernprozesse und vieles mehr). Kucharski erzählt in einer höchst informativen Weise die Geschichte der Mathematik in der Infektionsepidemiologie.

Das Buch führt fesselnd in die Infektionsepidemiologie ein. Es geht um deren Konzepte, Verfahren zur Datenerhebung und kritische Analysen abgeleiteter Handlungsanweisungen. Interaktionsgraphen sind ein zentrales Konzept zur Entwicklung effektiver Maßnahmen gegen eine Epidemie. Die abschließenden Kapitel beschreiben Ansätze zum Aufspüren von Ausbreitungswegen in Zeiten von Big Data und dem damit verbundenen Dilemma des Schutzes der Privatsphäre.

Das Buch spannt einen weiten, aber kohärenten thematischen Bogen. Es erzählt spannende und informative Geschichten zu Themen, die lebensnahe Fragen behandeln und deren Umsetzung in gute Wissenschaft aufzeigen. Obwohl es von mathematischem Denken lebt, braucht es keine Formeln. Die Graphiken sind klar und erhellend. Unübersichtliche Tabellen gibt es nicht. Adam Kucharski verbindet seine Geschichten mit wissenschaftlichen Arbeiten aus dem angesprochenen Sachgebiet. Beim Lesen erhält jeder einen gut strukturierten Zugang zur Originalliteratur.

Das Buch begeistert für eine transdisziplinäre Idee: eine Theorie zur Dynamik von Ereignissen – das Gesetz der Ansteckung. Dieses Projekt entwickelt sich aus interdisziplinärer Forschung.

Wo Kucharski sonst noch überall das Gesetz der Ansteckung am Werk sieht, möge jeder selbst nachlesen. Nur ein Punkt sei noch angesprochen: Verlage geben mit jedem Buch einer Idee die Chance sich in der Welt auszubreiten. Der Leser zeigt dabei einen spezifischen R-Wert. Welches R notwendig ist, um aus einem Buch einen Beststeller zu machen, kann mit dem von Kucharski vorgestellten Instrumentarium analysiert werden.

Für mich war das Buch ein großes Lesevergnügen. Spezialist/innen liefert es inspirierende Verbindungen zu scheinbar weitentfernten Themenfeldern. Was verbindet Infektionsepidemiolog/innen methodisch mit Sprach- oder Kulturwissenschaftler/innen? Für Einsteiger erläutert Kucharski wichtige infektionsepidemiologische Begriffe wie Reproduktionszahl, Infektionsmortalität, Inkubationszeit, Herdenimmunität, Impfrate etc. Nach der Lektüre lassen sich die gegenwärtigen Anstrengungen der Politik im Umgang mit der SARS-CoV-2 Epidemie besser verstehen.

Preis und Leistung stehen in einem hervorragenden Verhältnis. Das Buch verdient einen sehr weiten Leserkreis und wird weder Expert/innen noch Einsteiger/innen enttäuschen. ■

*Ulrich Mansmann,*
*Universität München,*
*mansmann@ibe.med.uni-muenchen.de*

* Open Access Dieser Artikel wird unter der Creative Commons Namensnennung 4.0 International Lizenz veröffentlicht, welche die Nutzung, Vervielfältigung, Bearbeitung, Verbreitung und Wiedergabe in jeglichem Medium und Format erlaubt, sofern Sie den/die ursprünglichen Autor(en) und die Quelle ordnungsgemäß nennen, einen Link zur Creative Commons Lizenz beifügen und angeben, ob Änderungen vorgenommen wurden. Die in diesem Artikel enthaltenen Bilder und sonstiges Drittmaterial unterliegen ebenfalls der genannten Creative Commons Lizenz, sofern sich aus der Abbildungslegende nichts anderes ergibt. Sofern das betreffende Material nicht unter der genannten Creative Commons Lizenz steht und die betreffende Handlung nicht nach gesetzlichen Vorschriften erlaubt ist, ist für die oben aufgeführten Weiterverwendungen des Materials die Einwilligung des jeweiligen Rechteinhabers einzuholen. Weitere Details zur Lizenz entnehmen Sie bitte der Lizenzinformation auf http://creativecommons.org/licenses/by/4.0/deed.de.

Funding note

Open Access funding enabled and organized by Projekt DEAL.

